# Treatment‐Specific Risk Scales for Identifying High‐Risk Patients With Poor Prognosis in Acute Ischemic Stroke: A Cohort Study From the National Neurological Medical Center of China

**DOI:** 10.1111/cns.70637

**Published:** 2025-11-05

**Authors:** Yi Xu, Shenyi Kuang, Shilin Yang, Jianfeng Luo, Chun Yu, Xiaocui Kang, Xiang Han, Qiang Dong

**Affiliations:** ^1^ Department of Neurology Huashan Hospital Fudan University Shanghai China; ^2^ School of Public Health Fudan University Shanghai China; ^3^ Intensive Care Unit of West Campus Huashan Hospital Fudan University Shanghai China; ^4^ Department of Neurology Fifth People's Hospital of Shanghai Fudan University Shanghai China; ^5^ Department of Neurology, State Key Laboratory of Medical Neurobiology, Huashan Hospital Fudan University Shanghai China; ^6^ State Key Laboratory of Medical Neurobiology and MOE Frontiers Center for Brain Science Fudan University Shanghai China

**Keywords:** acute ischemic stroke, acute‐phase prognostic model, nomogram, risk factors

## Abstract

**Aims:**

To develop and validate a user‐friendly scale for predicting acute‐phase adverse outcomes in acute ischemic stroke (AIS), thereby optimizing clinical management.

**Methods:**

This retrospective study enrolled AIS patients within 72 h of onset (excluding thrombectomy), stratified according to thrombolysis status to develop treatment‐specific prognostic models. The prognostic scale of AIS acute stage based on treatment stratification (PAIST) was developed using clinical variables, with discharge mRS as the primary endpoint, followed by external validation.

**Results:**

A total of 1971 AIS patients (437 thrombolyzed) were included. Both thrombolysis‐specific and non‐thrombolysis‐specific models incorporated core predictors (baseline NIHSS, deep vein thrombosis, neuron specific enolase, neutrophil percentage) but differed in cut‐off values and weightings. Additionally, the non‐thrombolysis‐specific model integrated three extra variables: age, fasting blood glucose, and serum potassium. External validation demonstrated PAIST outperformed the benchmark model (AUCs: thrombolysis group 0.759 vs. 0.698; non‐thrombolysis group 0.850 vs. 0.801; all *p* ≤ 0.05). PAIST‐based risk stratification effectively identified high‐risk patients, with poor prognosis rates of 76.92% (thrombolysis group) and 61.11% (non‐thrombolysis group).

**Conclusion:**

The PAIST scale is an effective and practical tool for acute‐phase prognostic risk stratification in AIS. Its treatment‐stratified design enables accurate risk assessment, thereby supporting individualized clinical decision‐making.

## Graphical abstract

The PAIST prognostic tool, developed through thrombolysis‐based stratification and externally validated, effectively identifies AIS patients at high risk of acute‐phase adverse outcomes by integrating multidimensional variables, demonstrating superior predictive performance to support clinical management.

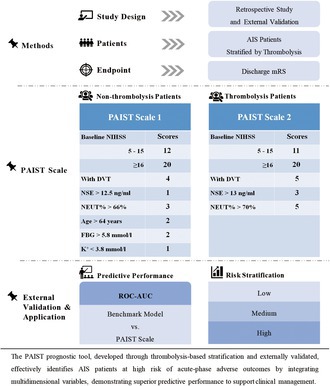

**Abbreviations**
AIS: acute ischemic stroke
AUC: area under the ROC curve
CI: confidence interval
DVT: deep vein thrombosis
FBG: fasting blood glucose
K^+^
: serum potassium
LAA: large‐artery atherosclerosis
mRS: modified rankin scale
NEUT%: neutrophils percentage
NIHSS: national institutes of health stroke scale
NSE: neuron‐specific enolase
OCSP: oxfordshire community stroke project
OR: odds ratio
PACI: partial anterior circulation infarction
PAIST: prognostic scale of AIS acute stage based on treatment stratification
POCI: posterior circulation infarction
PT: prothrombin time
RCS: restricted cubic spline
ROC: receiver operating characteristic
SOE: other determined etiologies
TOAST: trial of org 10,172 in acute stroke treatment

## 1 Introduction

Modifiable factors in the early stages of stroke are key to preventing early death and poor outcomes [1]. Identifying those factors and conducting targeted early monitoring or intervention can positively impact the prognosis of acute ischemic stroke (AIS) [2]. Current clinical practice still largely relies on physicians' subjective empirical judgment, which has certain limitations. Moreover, objective indicator‐based risk stratification tools have been proven to be more scientifically valuable than empirical decision‐making [3]. However, numerous single factors associated with poor prognosis in AIS have shown controversial effects on stroke outcome in different studies; therefore, integrating multiple factors to develop more precise prediction models is necessary. Additionally, most reported models lack specificity and often overlook available information during diagnosis and treatment (such as stroke etiology, treatment, and complications, or laboratory test indicators), which significantly impact outcomes [4, 5, 6]. Currently, there is a lack of widely applicable prognosis prediction models with high predictive power for AIS in the acute phase, which could assist in scientifically and efficiently managing AIS patients.

Notably, patients undergoing distinct therapeutic regimens during the acute stage of stroke exhibit marked heterogeneity. The direct construction of prognostic models for the entire AIS population fails to adequately capture disease‐specific characteristics and thus cannot enhance model accuracy. Herein, this study, conducted at China's National Neurological Medical Center, systematically integrates multidimensional clinical variables and employs rigorous statistical analyses. Using comprehensive clinical data from AIS patients in a strictly standardized expert‐led center, we developed and validated an efficient, widely applicable acute‐phase prognostic scale. This scale thereby serves as a reliable tool with substantial methodological merits and clinical utility for stroke prognosis prediction. Enrolled patients were stratified based on acute‐phase intravenous thrombolysis administration status, and separate prognostic models were established to offer novel insights into personalized therapeutic strategies during the acute phase.

## 2 Materials and Methods

### 2.1 Study Population

AIS patients discharged at the National Neurological Medical Center of China between 2015 and 2021 were included. The inclusion criteria were as follows: (1) patients diagnosed with AIS; (2) the interval between “last known normal time” and “admission time” at enrollment shall not exceed 72 h; (3) a pre‐stroke modified Rankin Scale [7] (mRS) score ≤ 2. Those who underwent arterial thrombectomy during the acute stage were excluded. The diagnostic and therapeutic approaches for the patients were in accordance with the current guidelines for stroke management [8, 9]. Formal written informed consent was procured from all patients. This study was approved by the Ethics Committee and was in strict accordance with the Declaration of Helsinki.

### 2.2 Data Collection and Outcome

Complete baseline clinical characteristics (65 items) were collected for inpatients with AIS, including demographic information at admission, history of previous illnesses and medication use, and laboratory results. Laboratory indicators met three criteria: (1) all are routine clinical tests; (2) standardized collection procedures (first morning blood upon admission) were applied; and (3) certified automated testing systems were used. Moreover, stroke‐related classification and scoring features were included (Figures S1 and S2). The severity of AIS was assessed using the baseline National Institutes of Health Stroke Scale (NIHSS) score [10], categorized as mild (≤ 4), moderate (5–15), and severe (≥ 16) according to the widely adopted three‐tier classification in stroke, which reflects key clinical thresholds for neurological deficits and ensures clinical operability, robustness, and comparability [11, 12]. Given that most recovery in AIS occurs within 4 days and predicts long‐term functional outcome [13], the primary outcome of this study was the degree of neurological deterioration during hospitalization, as assessed by the discharge mRS. The mRS, a standard stroke functional assessment tool, ranges from 0 to 6 [7] (Table S1). Each patient's mRS score was determined at the time of discharge by two senior neurologists blinded to clinical data to ensure objectivity. Poor prognosis was defined as a discharge mRS > 2, a clinically validated cutoff widely used in stroke studies [3, 14].

### 2.3 Study Grouping

**FIGURE 1:**
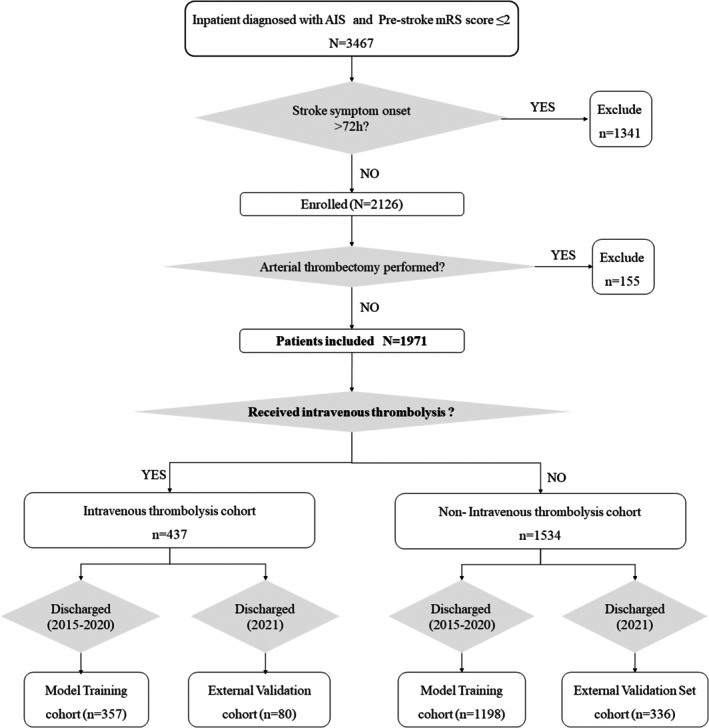
Study population grouping and enrollment flow. From 3467 AIS patients with pre‐stroke mRS ≤ 2, 1341 (onset > 72 h) and 155 (receiving thrombectomy) were excluded, leaving 1971 patients for analysis. These patients were stratified by intravenous thrombolysis status, and further divided into model training cohorts (discharged between 2015 and 2020) and external validation cohorts (discharged in 2021).

The enrolled patients were divided into two cohorts based on the administration of intravenous thrombolytic therapy (Figure 1): the thrombolysis cohort (*n* = 437) and the non‐thrombolysis cohort (*n* = 1534), and separate predictive models for adverse outcomes were developed and validated for each cohort. The discharge date was utilized as the criterion for cohort dataset assignment. Patients discharged between January 1, 2015, and December 31, 2020 comprised the training dataset (*n* = 1555) for model development, and those discharged between January 1, 2021, and December 31, 2021 constituted the external validation dataset (*n* = 416) to evaluate the models' predictive accuracy.

### 2.4 Model Construction and Validation

The study employed a rigorous group‐specific independent strategy, with completely independent modeling processes for thrombolytic and non‐thrombolytic populations: model development was conducted using their respective training sets, and validation was performed on independent validation sets with no overlap with the modeling populations (Figure S3).

For each cohort, a three‐stage feature selection process was applied to the training dataset. First, variables with statistical significance (two‐tailed *p* < 0.05) in univariate analysis were selected for further evaluation. Then, multivariable logistic regression with stepwise variable selection (combining forward and backward methods) was performed, retaining features with significant (*p* < 0.1). Ultimately, a logistic regression model with restricted cubic splines (RCS) and three knots for each continuous feature was used to estimate the risk of poor prognosis, forming the basis of the continuous models. These models also characterized associations between continuous variables and the risk of poor AIS outcomes within each cohort.

To enhance model robustness, RCS analysis with three knots was applied to continuous variables in the continuous model to identify critical cut‐off values. These values were defined as the point where odds ratios (OR) crossed 1. All cut‐off calculations were adjusted for other variables in the model. These categorized variables were then incorporated into logistic regression models to derive regression coefficients (i.e., scores) for building cohort‐specific categorical models.

For clinical practicality, each variable's coefficient in the categorical model was divided by five and rounded to the nearest integer, ultimately forming the Prognostic scale of AIS acute stage based on Treatment stratification (PAIST Scale).

Receiver operating characteristic (ROC) curves and the area under the ROC curve (AUC) were evaluated for all models, and the performance of each scale, including the PAIST, was compared between the two independent validation cohorts using the Delong test. Given that the “baseline NIHSS score” is a well‐recognized core prognostic factor for AIS, and studies have noted that a baseline NIHSS score ≥ 16 is significantly associated with increased risk of death or severity outcomes [10, 15]. Therefore, we constructed a separate benchmark model for each cohort, based on the “baseline NIHSS score” (a continuous variable), which was used as a benchmark model for predicting outcomes and to statistically compare the AUC values of the aforementioned models to determine their significance in this study. In the external validation sets of the two cohorts, statistical comparisons of AUC values were performed between these benchmark models and the three new models (the Continuous model, Categorical model, and PAIST Scale) to evaluate the predictive efficacy of the scales.

To further validate the PAIST Scale's risk stratification capability, patients were stratified into three risk tiers based on the predicted probability of adverse prognosis derived from the PAIST Scale score: low risk (risk probability ≤ 0.1), medium risk (risk probability > 0.1 and ≤ 0.5), and high risk (risk probability > 0.5). The actual incidence of mRS > 2 at discharge within the validation cohort was subsequently calculated for each risk cohort.

### 2.5 Statistical Analysis

Baseline characteristics were compared between groups as well as the training and validation datasets (Table S2). Statistical differences for categorical variables were evaluated using the chi‐squared test. For continuous variables, normality was tested using the Shapiro–Wilk test: normally distributed variables were analyzed with Student's *t*‐test, while non‐normally distributed variables were compared using the Mann–Whitney *U* test. A two‐sided *p*‐value < 0.05 was considered statistically significant. Missing data were imputed in each group with the multivariate imputation by chained equations methods. All statistical analyses were performed using R, version 4.1.2.

## 3 Results

### 3.1 Baseline Characteristics

A total of 1971 patients were enrolled in this study (Figure 1). In the non‐thrombolysis cohort, the mean age was 63.3 ± 13.1 years, with a male predominance (73.5%). At hospital discharge, 30.2% of patients had a mRS score > 2, and the mortality rate was 1.76%. Based on baseline NIHSS score, 58.22% were classified as having mild strokes (score ≤ 4), 35.72% as moderate strokes (score 5–15), and 6.06% as severe strokes (score ≥ 16). The most common Oxfordshire Community Stroke Project (OCSP) classification was Partial Anterior Circulation Infarction (PACI), accounting for 51.1% of cases, followed by Posterior Circulation Infarction (POCI) at 30.1%. For the Trial of Org 10,172 in Acute Stroke Treatment (TOAST) classification, the majority were Large‐Artery Atherosclerosis (LAA, 37.3%), while Other Determined Etiologies (SOE, 3.1%) were the least frequent. In contrast, the thrombolysis cohort had a higher mean age (66.8 ± 11.8 years) compared to the non‐thrombolysis cohort (*p* < 0.001), with a male prevalence of 68.9%. Most patients in this subgroup had moderate strokes (51.94%). The most prevalent OCSP subtype was PACI (59.5%), followed by POCI (19.9%). For TOAST classification, LAA (36.1%) was the most common, and SOE (1.6%) was the least frequent (Table 1, Figure S4).

**TABLE 1:** Basic characteristics between the non‐thrombolytic and the thrombolytic cohort.

	Non‐thrombolytic cohort (*N* = 1534)	Thrombolytic cohort (*N* = 437)	*p*
Outcome event, *n* (%)			
Discharge mRS > 2	463 (30.2%)	151 (34.6%)	0.093
Mortality	27 (1.76%)	19 (4.35%)	0.003
General data, mean (SD)			
Number of male (%)	1128 (73.5%)	301 (68.9%)	0.063
Age (year)	63.3 (13.1)	66.8 (11.8)	< 0.001
BMI (kg/m^2^)	26.0 (17.1)	25.8 (15.8)	0.768
Days from onset to hospital admission	1.58 (0.80)	0.32 (0.48)	< 0.001
SBP on admission (mmHg)	151 (22.7)	152 (22.4)	0.322
DBP on admission (mmHg)	84.9 (12.3)	82.4 (12.9)	< 0.001
Individual with DVT (%)	463 (30.2%)	136 (31.1%)	0.751
Medical history, *n* (%)			
Antidiabetic medication use	301 (19.6%)	102 (23.3%)	0.102
Antihypertensive medication use	722 (47.1%)	251 (57.4%)	< 0.001
Lipid‐lowering medication use	189 (12.3%)	59 (13.5%)	0.566
Previous stroke	103 (6.71%)	34 (7.78%)	0.505
Previous myocardial infarction	42 (2.74%)	12 (2.75%)	1
History of atrial fibrillation	200 (13.0%)	88 (20.1%)	< 0.001
History of hypertension	680 (44.3%)	216 (49.4%)	0.067
History of diabetes mellitus	264 (17.2%)	84 (19.2%)	0.367
History of smoking	1137 (74.1%)	325 (74.4%)	0.965
History of alcohol consumption	910 (59.3%)	269 (61.6%)	0.432
Stroke‐related scores, *n* (%)			
Essen stroke risk score			0.159
Low risk (Essen ≤ 2)	995 (64.86%)	264 (60.41%)	
Moderate risk (Essen 3–6)	536 (34.94%)	173 (39.59%)	
High risk (Essen ≥ 7)	3 (0.20%)	0 (0.00%)	
Baseline NIHSS score, *n* (%)			< 0.001
Mild stroke (NHISS ≤ 4)	893 (58.22%)	167 (38.22%)	
Moderate stroke (NHISS 5–15)	548 (35.72%)	227 (51.94%)	
Severe stroke (NHISS ≥ 16)	93 (6.06%)	43 (9.84%)	

Abbreviations: BMI, body mass index; DBP, diastolic blood pressure; DVT, deep venous thrombosis; mRS, modified rankin scale; NIHSS, national institutes of health stroke scale; SBP, systolic blood pressure.

### 3.2 Risk Factors Identification

Based on the training dataset from two cohorts, we have ascertained the significant risk factors correlated with poor prognosis in AIS by univariate (Tables S3 and S4) and multivariate logistic regression analysis (Table S5), respectively. Within two distinct cohorts, the identified risk factors associated with an adverse prognosis included: baseline NIHSS scores, combined deep vein thrombosis (DVT), levels of neuron‐specific enolase (NSE, ng/ml), and neutrophils percentage (NEUT%). Additionally, in the non‐thrombolysis cohort, significant predictors also comprised age, prothrombin time (PT, in seconds), fasting blood glucose concentration (FBG, mmol/L), and serum potassium levels (K^+^, mmol/L). Subsequently, prognostic models were independently constructed for both cohorts, designated as continuous models, and graphically represented in the form of nomograms (Figure S5).

### 3.3 PAIST Scale Construction

**FIGURE 2:**
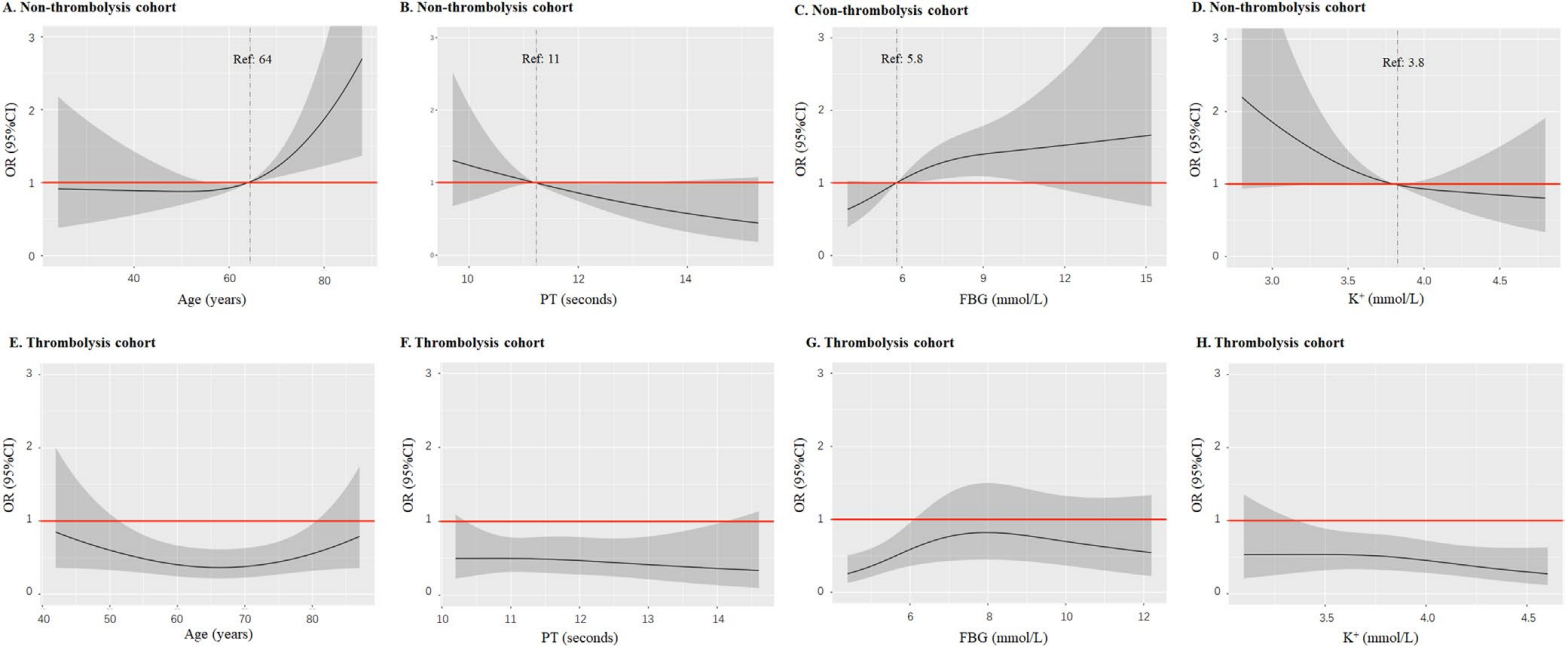
Nonlinear Associations of Differential Clinical Indicators with Poor Stroke Outcomes between the Two Cohorts. This figure presents associations analyzed using restricted cubic spline with three knots. Due to inherent differences in data distribution, *x*‐axis scales vary between cohorts; thus, interpretation should focus on curve‐derived association trends rather than absolute numerical positions. Reference thresholds were determined via restricted cubic splines analysis, identifying inflection points where OR = 1. OR estimates were adjusted for covariates: Non‐thrombolysis cohort (baseline NIHSS ≤ 4, DVT‐free, NSE = 12.5 ng/ml, NEUT% = 66%, PT = 11 s, FBG = 5.8 mmol/L, K^+^ = 3.8 mmol/L, age = 64 years) and thrombolysis cohort (baseline NIHSS 5–15, DVT‐free, NSE = 13 ng/mL, NEUT% = 70%), with analyzed variables excluded. Solid red line: Reference line (OR = 1); Gray shaded area: 95% confidence interval of the estimated OR; Dashed vertical line: Cutoff value where OR = 1. CI, confidence interval; DVT, deep vein thrombosis; FBG, fasting blood glucose; K^+^, serum potassium; NEUT%, neutrophil percentage; NIHSS, National Institutes of Health Stroke Scale; NSE, neuron‐specific enolase; OR, odds ratio; PT, prothrombin time; Ref, reference value.

Cut‐off values of continuous indicators associated with poor prognosis in continuous models were derived via ROC analysis. Among these, NSE and NEUT% emerged as significant predictors of adverse prognosis in both cohorts, though their optimal cutoff values differed between cohorts. In the non‐thrombolysis cohort, the risk of adverse prognosis was minimal when NSE < 12.5 ng/mL (Figure S6A). By contrast, in the thrombolysis cohort, NSE < 13 ng/mL correlated with a reduced risk of adverse prognosis (Figure S6B). For NEUT%: In the non‐thrombolysis cohort, levels > 66% were linked to a significantly higher risk of poor prognosis (Figure S6C). In the thrombolysis cohort, no significant NEUT% cut‐off for adverse prognosis was identified, as the 95% confidence interval (CI) of the OR in RCS analysis included 1. Despite this, a cut‐off of 70% was used for model construction, corresponding to the point where the OR crossed 1 (Figure S6D). Additionally, in the non‐thrombolysis cohort, the following factors were associated with an increased risk of poor AIS prognosis: age > 64 years, PT < 11 s, FBG > 5.8 mmol/L, and serum K^+^ < 3.8 mmol/L (Figure 2A–D). No such association with increased adverse prognosis risk was observed in the thrombolysis cohort for these factors (Figure 2E–H). Categorical models were constructed using optimal cut‐off values from continuous parameters (Table S6), which were then refined to develop the PAIST Scale (Table 2). Notably, PT was excluded from the non‐thrombolysis cohort's PAIST Scale due to its negligible regression coefficient (to 0.2) when modeled as a categorical variable.

**TABLE 2:** Prognostic scale of AIS acute stage based on treatment stratification (PAIST scale).

PAIST Scale 1	
	Scores
Baseline NIHSS	
5–15	**12**
≥ 16	**20**
With DVT	**4**
NSE > 12.5 ng/ml	**1**
NEUT% > 66%	**3**
Age > 64 years	**2**
FBG > 5.8 mmol/L	**2**
K^+^ < 3.8 mmol/L	**1**

PAIST Scale 2	
	Scores
Baseline NIHSS	
5–15	**11**
≥ 16	**20**
With DVT	**5**
NSE > 13 ng/mL	**3**
NEUT% > 70%	**5**

*Note:* PAIST Scale 1 is for the non‐Thrombolysis patients; PAIST Scale 2 is for Thrombolysis patients.

Abbreviations: AIS, Acute Ischemic Stroke; DVT, Deep Vein Thrombosis; FBG, Fasting Blood Glucose; K^+^, Serum Potassium; NEUT%, Neutrophil Percentage; NSE, Neuron Specific Enolase.

### 3.4 PAIST Scale Validation

**FIGURE 3:**
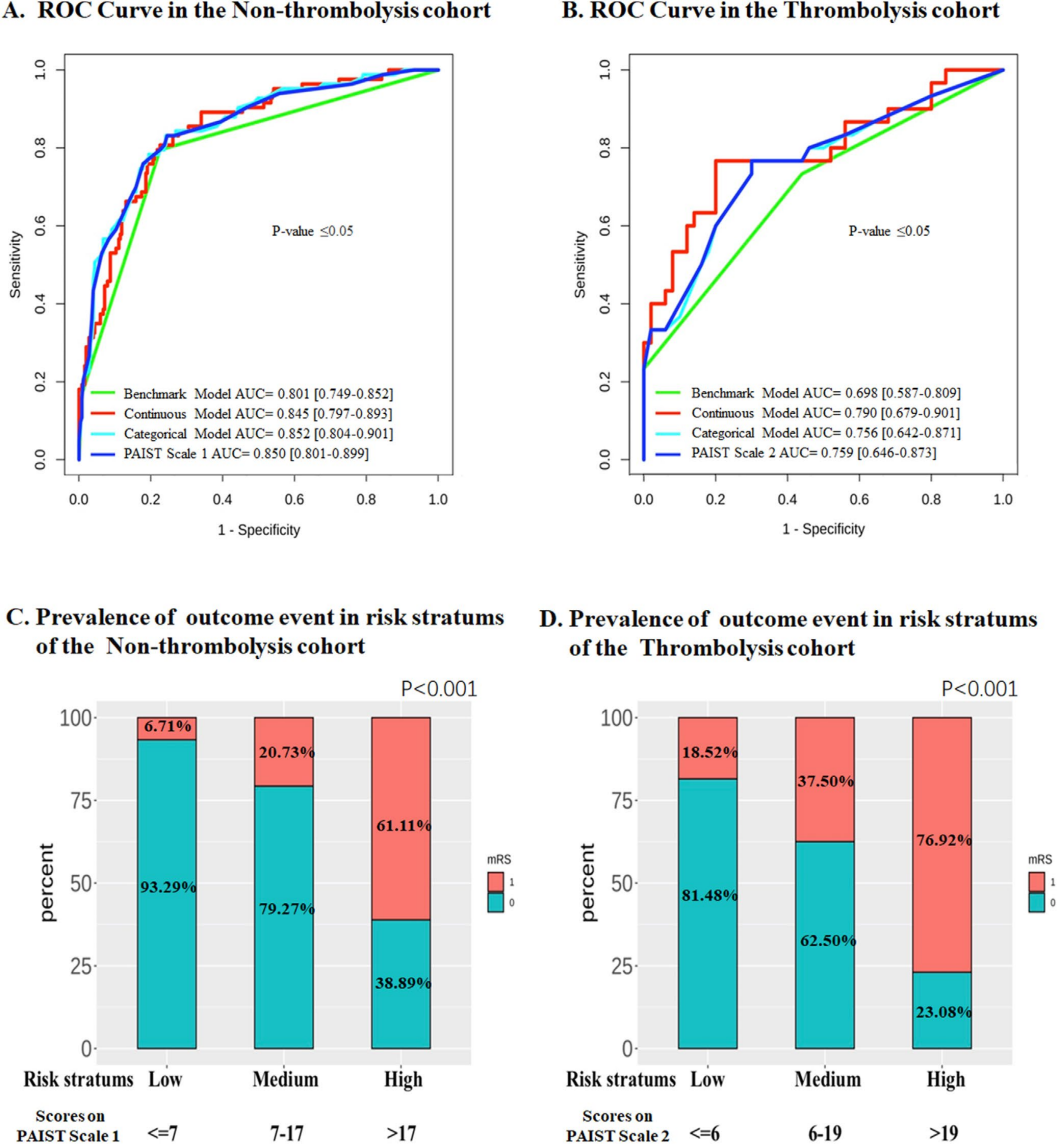
Prognostic predictive power of the PAIST scale. The PAIST Scale (Prognostic Scale of AIS Acute Stage Based on Treatment Stratification) includes two subscales: PAIST Scale 1 is used for non‐thrombolysis patients, and PAIST Scale 2 for thrombolysis patients. mRS 1, mRS > 2 at discharge (poor outcome); mRS 0, mRS ≤ 2 at discharge (favorable outcome); Risk strata, risk of poor prognosis. (A, B) ROC curves demonstrate the prognostic performance of each model in the external validation cohorts. The *x*‐axis representing 1‐specificity, and the *y*‐axis representing sensitivity. Compared with the benchmark model (continuous baseline NIHSS score), all other models showed statistically significant differences (DeLong test, *p* ≤ 0.05). (C, D) show the actual incidence (%) of adverse outcomes in the two cohorts, which were stratified by PAIST risk levels (low, medium, high). Bars represent the percentage of patients with adverse (red) or favorable (green) outcomes; numerical values denote exact proportions.

Utilizing external validation datasets from both cohorts, the prognostic predictive capabilities of the Continuous Models, Categorical Models, and the PAIST Scale were assessed. The AUC values for the PAIST Scale in both cohorts were significantly superior to those of the Benchmark Model (0.850 for the non‐thrombolysis cohort; 0.759 for the thrombolysis cohort) (Figure 3A,B). Furthermore, no significant reduction in the predicting power of the PAIST Scale was observed, when compared with both Continuous and Categorical Models (*p* = 0.53 and *p* = 0.12 for non‐thrombolysis; *p* = 0.18 and *p* = 0.29 for thrombolysis cohorts, respectively).

A risk stratification scheme was subsequently developed based on the total scores of the PAIST Scale (Table 3). To further assess the stratification capability of the PAIST Scale for adverse prognosis, the non‐thrombolysis cohort was stratified into low (*n* = 164), moderate (*n* = 82), and high (*n* = 90) risk groups based on the “Non‐Thrombolysis PAIST scale” for the external validation dataset. The actual number of patients experiencing poor prognosis in these groups were 11, 17, and 55, respectively (Figure 3C). For the thrombolysis cohort, risk stratification using the “Thrombolysis PAIST scale” yielded low (*n* = 27), moderate (*n* = 40), and high (*n* = 13) risk groups. The corresponding numbers of patients with poor prognosis were 5, 15, and 10, respectively (Figure 3D). In both cohorts, the moderate‐risk group exhibited a significantly higher incidence of poor prognosis compared to the low‐risk group (*p* < 0.001), and conversely, a significantly lower incidence relative to the high‐risk group (*p* < 0.001).

**TABLE 3:** Risk stratification for the PAIST scale.

Risk stratification	Risk probability of poor prognosis	Score on PAIST scale 1	Score on PAIST scale 2
Low risk	5%	4	2
	10%	7	6
Moderate risk	30%	13	14
	50%	17	19
High risk	70%	20	23
	90%	26	31
	95%	30	/

*Note:* PAIST Scale 1 is for the non‐Thrombolysis patients; PAIST Scale 2 is for Thrombolysis patients. The color coding is for visual clarification only: green, low risk; orange, moderate risk; red, high risk.

Abbreviation: PAIST scale, prognostic scale of AIS acute stage based on treatment stratification.

## 4 Discussion

Leveraging comprehensive clinical data from hospitalized patients with AIS, this study developed the novel PAIST Scale for predicting poor prognosis in the acute phase. Recognizing the inherent heterogeneity between thrombolytic and non‐thrombolytic populations, distinct predictive models were developed to capture their unique prognostic profiles, resulting in two respective subscales (PAIST 1 and PAIST 2). To enhance clinical utility, the continuous nomogram‐based prediction models were further simplified into the PAIST scoring scale. External validation showed that the PAIST Scale retained predictive performance comparable to the continuous nomogram models, while significantly outperforming the benchmark model, even when substantial baseline differences existed between the training and validation cohorts (Table S2). Particularly noteworthy is its superior performance in identifying high‐risk individuals among patients with mild to moderate stroke (baseline NIHSS score < 16), enabling clinicians to perform precise risk stratification and deliver targeted monitoring and interventions during the critical acute phase. Furthermore, the study revealed differences in risk factors for poor acute prognosis between the two cohorts. Beyond differences in the factors themselves, the optimal cut‐off values and prognostic weights of shared indicators also differed, which further validates the value of our stratified modeling strategy.

These differential risk profiles provide important clinical implications. Similar risk factors for adverse outcomes were observed in both cohorts, including elevated baseline NIHSS score, concomitant DVT, increased NSE levels, and elevated neutrophil percentage (NEUT%). The baseline NIHSS score is widely considered an important determinant of AIS prognosis [16]. DVT, a serious complication in AIS patients, often results from limb immobility or paralysis [17]. Although D‐dimer levels are generally thought to indicate the occurrence of DVT, Ha et al. reported that DVT in Asian stroke patients correlates with stroke severity, not D‐dimer levels [18], and the value of D‐dimer levels in indicating DVT in AIS patients was low, which may explain the exclusion of D‐dimer from the PAIST Scale. NSE, as a specific marker of brain neuronal damage, aids in cranial injury severity assessment and stroke outcome prediction [19]. In addition, inflammatory responses and blood–brain barrier disruption were associated with poor stroke outcomes [20, 21], and this association was also very evident in patients who experienced hemorrhagic transformation after recombinant tissue plasminogen activator thrombolysis [22]. Neutrophils are commonly used as inflammatory markers and affect vascular remodeling during the recovery period of stroke [23], which has important functional significance for brain repair after stroke. Moreover, NEUT% was higher in acute cerebral infarction patients than in those with transient ischemic attack [24], further suggesting that the NEUT% was positively correlated with the severity of stroke. High neutrophil counts during the acute stage of stroke were more sensitive as predictive factors for poor stroke outcomes [25]. Multiple studies have also suggested that neutrophils were significantly associated with prognosis in the acute stage of stroke, such as the CNSRIII study [26], and high levels of NEUT% were also associated with an increased risk of new stroke, composite events, and ischemic stroke [27].

Building on these shared risk factors, comparison between cohorts revealed distinct patterns. When comparing the same adverse outcome risk factors between the two cohorts, higher cut‐off values for NSE and NEUT% were observed in the thrombolysis cohort. These differences may reflect not only thrombolysis‐related pathophysiological changes but also greater baseline disease severity in this cohort. Additionally, these differences might be associated with the different baseline serological examination times between the two cohorts, as thrombolysis patients were tested within 24 h after admission, whereas non‐thrombolysis patients were tested between 36 and 48 h.

Beyond these common factors, non‐thrombolysis patients exhibited a broader range of risk determinants. Among non‐laboratory indicators, advanced age, a well‐established prognostic factor in AIS [16], was associated with adverse outcomes only in non‐thrombolysis patients, consistent with studies indicating that older age exacerbates stroke‐related complications and mortality [28, 29, 30]. Although age has traditionally been considered a relative contraindication for thrombolysis, the findings of this study indirectly suggested that thrombolysis might mitigate the risk of adverse outcomes in elderly patients to some extent. Thus, in the thrombolysis cohort of the PAIST scale, age was not a high‐risk factor for adverse outcomes, but this requires further validation. Regarding laboratory parameters, non‐thrombolysis patients were susceptible to adverse outcomes not only from inflammation factors but also from elevated FBG and hypokalemia. Stroke patients with robust collateral circulation were more likely to achieve a favorable prognosis, and those with better cerebral collateral circulation tended to exhibit lower baseline blood glucose levels [31], and FBG was positively associated with stroke risk [32] and predicted progressive stroke within 48 h of onset in AIS patients [33]. However, the benefit of stringent glycemic control during thrombolysis requires higher‐level evidence [34]. In line with our results, the association between elevated FBG and increased risk of adverse outcomes was significant only in the non‐thrombolysis cohort, suggesting that the criteria for glycemic control may be moderately relaxed in the thrombolysis patients. Similarly, higher potassium intake was linked to a reduced risk of all ischemic strokes and all‐cause mortality [35, 36]. Herein, hypokalemia in non‐thrombolysis patients was associated with adverse outcomes during the acute stage of stroke; consistently, the CNSRIII study also confirmed hypokalemia was correlated with adverse outcomes and a heightened risk of death at 3 months and 1 year after stroke [37]. The optimal serum potassium cut‐off for poor prognosis was suggested as 3.7 mmol/L [38], which was close to the 3.8 mmol/L derived from this study.

In terms of baseline characteristics, a higher proportion of thrombolysis patients exhibited adverse outcomes (discharge mRS > 2), which might be related to the higher baseline NIHSS scores and older average age in this cohort. The risk relationship diagram (Figure 2) revealed a near‐linear association between FBG, serum potassium levels and adverse outcomes in the acute stage of AIS, which differs from the widely held clinical belief that both high and low levels of these parameters are detrimental. This discrepancy might be related to the study population's characteristic distribution, as hypoglycemia was more aggressively managed in clinical practice than hyperglycemia, leading to a smaller sample size of fasting hypoglycemia, with most subjects' FBG levels concentrated in a certain range. A similar reason also resulted in a smaller sample size of subjects with potassium levels above the normal range in this study population. This study identified critical values for continuous indicators associated with adverse outcomes, representing the points at which the likelihood of adverse outcomes was highest for that indicator compared to other reference ranges. However, these findings do not advocate for arbitrary blood glucose reduction or potassium supplementation in clinical practice. Prompt detection and correction of hypoglycemia are vital to prevent neurological deficits and neuronal damage [8]. Furthermore, potassium supplementation must consider patients' renal function, as excessive potassium can cause fatal complications in patients with impaired renal function [39].

When compared to existing prognostic models, the PAIST Scale demonstrates several innovative advantages in AIS acute‐stage prognostic prediction. Methodologically, it adopts multi‐dimensional parameter selection and objective data‐driven modeling [3], thereby minimizing subjective selection bias. This approach allows for more systematic identification of stroke prognosis determinants, ensuring both the scientific rigor of model construction and the objectivity of threshold determination, while addressing limitations of single‐biomarker approaches. In contrast, models like SPAN‐100 rely solely on age and NIHSS; while operationally simple, they compromise accuracy through oversimplification [40]; the DRAGON score, though evidence‐based, faces limitations in the availability of comprehensive biomarker and comorbidity data, as well as consistency in imaging interpretation [41]; PREMISE suffers from sample selection bias [42]. Furthermore, the PAIST Scale incorporates a stratified modeling strategy, accounting for treatment differences by developing separate prognostic models for thrombolytic and non‐thrombolytic populations. This approach outperforms traditional models like iScore and Revised iScore, which do not fully account for the impact of thrombolytic therapy on prognosis [2, 43]. In terms of prediction dimensions, the PAIST Scale focuses on functional outcomes, aligning more closely with clinical decision‐making needs compared to models predicting only mortality (e.g., mSOAR [44], PREMISE [42], Get With the Guidelines [15]) or hemorrhagic complications (e.g., SEDAN [45]). Notably, the PAIST Scale was developed using Chinese population data, addressing the limitations of previous Western‐based models. While maintaining high predictive performance, it utilizes routinely available clinical data without requiring advanced imaging or specialist interpretation. By balancing scientific rigor with practicality, it simplifies data requirements compared to models requiring numerous variables or specialized expertise [43, 44, 45], demonstrating particular utility in resource‐limited settings.

Several limitations of this study warrant consideration. First, as a single‐center retrospective investigation, although leveraging data from the National Neurological Medical Center of China ensured high data quality, reliability, and consistency through standardized guideline adherence, the sample size remains relatively limited. This constraint was particularly evident during external validation, where the thrombolysis cohort was small (*n* = 80), potentially reducing statistical power. Specifically, the DeLong test employed may have had diminished sensitivity in detecting subtle differences in AUC values between models under such sample size constraints, possibly leading to underestimation of the distinction between the PAIST scale and the benchmark model. Nevertheless, even within this limited validation cohort, the PAIST scale demonstrated robust risk stratification ability, providing crucial preliminary evidence of its generalizability. Second, the use of discharge mRS as the primary endpoint, while inferior to 3‐month mRS in evaluating functional outcomes, was determined by the research objectives and the retrospective design. Although this endpoint may be influenced by short‐term factors such as length of hospitalization duration and acute‐phase complications, it directly reflects neurological status at acute‐phase completion. This measure aligns closely with key clinical decision points, including rehabilitation timing and discharge planning. Moreover, standardized assessment protocols ensured scoring reliability. While not substituting for long‐term endpoints, it offers distinct clinical value in acute‐phase prognosis evaluation. Finally, the trichotomization of baseline NIHSS scores in the PAIST scale may result in loss of predictive information inherent in continuous variables. However, this categorization follows conventional stroke research standards, balancing clinical practicality with operational feasibility. Modeling results indicated that baseline NIHSS score carried substantial predictive weight—a finding consistent with clinical experience. This also suggests that among patients with severe baseline deficits (NIHSS ≥ 16) who did not receive thrombolysis, the contribution of other PAIST predictors may be limited. It should be emphasized that PAIST's primary value lies in significantly improving prediction of acute‐phase adverse outcomes in mild‐to‐moderate stroke patients compared to traditional reliance on NIHSS alone. By integrating multidimensional indicators, it enables more refined risk stratification. Validation results confirm that this multi‐parameter integration enhances discriminative performance, highlighting its clinical utility in mild‐to‐moderate stroke populations.

Future research should validate the PAIST scale in multicenter prospective cohorts. Concurrently collecting both discharge and 3‐month mRS will be crucial to compare their predictive utility and refine long‐term forecasts, and clarify the correlation between acute‐phase and long‐term prognostic trends. Furthermore, leveraging the practicality of PAIST, an embedded scoring system could be developed within electronic health records to automate baseline data extraction, enable real‐time risk evaluation, and support intelligent early warning, thereby enhancing clinical decision‐making and advancing precision management in acute stroke care.

## 5 Conclusion

The PAIST scale, developed via a treatment stratified approach, demonstrates significant advantages in prognostic prediction and clinical applicability for AIS in the acute phase. By integrating multidimensional clinical variables and adopting stratified modeling, it enables individualized risk prediction for both thrombolyzed and non‐thrombolyzed AIS patients. Through its robust discriminative ability and generalizability, the scale effectively identifies patients at high risk of acute‐phase adverse outcomes among those with mild‐to‐moderate AIS. The implementation of the PAIST scale is expected to provide quantitative support for formulating acute‐phase treatment strategies and hold potential value for advancing precise and individualized clinical management of stroke.

## Ethics Statement

This study was approved by the Ethics Committee and was in strict accordance with the Declaration of Helsinki.

## Conflicts of Interest

The authors declare no conflicts of interest.

## Supporting information


**Table S1:** Modified Rankin Scale (mRS) grades and Corresponding Disability Levels.
**Table S2:** Comparison of Baseline Characteristics Between Thrombolysis and Non‐Thrombolysis Cohorts Across Training and External Validation Datasets.
**Table S3:** Significant Indicators from Univariate Analysis in the Thrombolysis Cohort.
**Table S4:** Significant Indicators from Univariate Analysis in the Non‐thrombolysis Cohort.
**Table S5:** Multivariate Regression Analysis of Differential Indicators Between the Thrombolysis and the Non‐thrombolysis Cohorts.
**Table S6:** The Categorical Model and Corresponding Integral Metrics with their Respective Score Values.
**Figure S1:** Variables Collected in the Study: Non‐laboratory Indicators.
**Figure S2:** Variables Collected in the Study: Laboratory Indicators. ESR, Erythrocyte Sedimentation Rate; NT‐ProBNP, N‐Terminal Pro‐B‐Type Natriuretic Peptide; eGFR, Estimated Glomerular Filtration Rate.
**Figure S3:** Development and Validation of the PAIST Scale for Prognostic Risk Stratification in Ischemic Stroke Patients. RCS, restricted cubic splines; PAIST Scale, Prognostic Scale of AIS Acute Stage Based on Treatment Stratification; AUC‐ROC, area under the receiver operating characteristic curve. Analyses were performed separately in thrombolysis and non‐thrombolysis cohorts. Development phase (model training sets, patients discharged between 2015 and 2020): Risk features were selected via univariate and multivariate regression (excluding collinear or insignificant variables). Continuous variables analyzed using 3‐knot RCS to identify cut‐offs (OR = 1, adjusted for covariates), then categorized and incorporated into logistic regression. Coefficients were standardized to derive the PAIST scale, which stratifies patients into low (< 10%), moderate (10%–50%), and high (> 50%) risk groups for poor prognosis. Validation phase (external validation sets, patients discharged in 2021): The predictive performance of the PAIST Scale was evaluated against a baseline NIHSS‐based benchmark model (via DeLong test for AUC‐ROC comparison), and its risk stratification was further validated.
**Figure S4:** Distribution of Stroke Subtypes in Subgroup Populations. (A) OCSP, Oxfordshire Community Stroke Project; LACI, Lacunar Infarct; PAIC, Partial Anterior Circulation Infarct; POCI, Posterior Circulation Infarct; TACI, Total Anterior Circulation Infarct; (B) TOAST, Trial of Org 10,172 in Acute Stroke Treatment; SAO, Small Artery Occlusion; LAA, Large‐Artery Atherosclerosis; CE, Cardioembolism; SOE, Other Determined Etiology; SUE, Undetermined Etiology.
**Figure S5:** Nomograms of Continuous Models for Predicting Poor Prognosis Risk in AIS.
Variables included in the nomograms and their definitions: Baseline NIHSS (0 = NIHSS ≤ 4; 1 = NIHSS 5–15; 2 = NIHSS ≥ 16); DVT (deep vein thrombosis, 0 = No, 1 = Yes); NIHSS, National Institutes of Health Stroke Scale; NSE, Neuron Specific Enolase; NEUT%, Neutrophil Percentage; PT, Prothrombin Time; FBG, Fasting Blood Glucose; Serum K^+^, Serum Potassium. Each variable is assigned points value, and the total points correspond to both a predicted risk probability (on a 0–10 scale) and an absolute risk (0–100%). The bottom axis indicates risk stratification from low to high.
**Figure S6:** Consistent Nonlinear Associations of Clinical Indicators With Poor Stroke Outcomes Across Two Cohorts. This figure presents associations analyzed using restricted cubic spline with 3 knots. OR, odds ratio; CI, confidence interval; NIHSS, National Institutes of Health Stroke Scale; DVT, Deep Vein Thrombosis; NSE, Neuron‐Specific Enolase; NEUT%, Neutrophil Percentage; PT, Prothrombin Time; FBG, Fasting Blood Glucose; K^+^, Serum Potassium, Ref, Reference Value. Due to inherent differences in data distribution, *x*‐axis scales vary between cohorts; thus, interpretation should focus on curve‐derived association trends rather than absolute numerical positions. Reference thresholds were determined via restricted cubic splines analysis, identifying inflection points where OR = 1. OR estimates were adjusted for covariates: non‐thrombolysis cohort (baseline NIHSS ≤ 4, DVT‐free, NSE = 12.5 ng/mL, NEUT% = 66%, PT = 11 s, FBG = 5.8 mmol/L, K^+^ = 3.8 mmol/L, age = 64 years) and thrombolysis cohort (baseline NIHSS 5–15, DVT‐free, NSE = 13 ng/mL, NEUT% = 70%), with analyzed variables excluded. Solid red line: Reference line (OR = 1); Gray shaded area: 95% confidence interval of the estimated OR; Dashed vertical line: cutoff value where OR = 1.

## Data Availability

The data that support the findings of this study are available from the corresponding author upon reasonable request.
