# Supplementary material for: Treatment‐Specific Risk Scales for Identifying High‐Risk Patients With Poor Prognosis in Acute Ischemic Stroke: A Cohort Study From the National Neurological Medical Center of China
Source: CNS Neurosci Ther. 2025 Nov 5;31(11):e70637. doi: 10.1111/cns.70637 (PMC12588881; doi:10.1111/cns.70637)
Supplement: Supplementary file 1 — Table S1: Modified Rankin Scale (mRS) grades and Corresponding Disability Levels. Table S2: Comparison of Baseline Characteristics Between Thrombolysis and Non‐Thrombolysis Cohorts Across Training and External Validation Datasets. Table S3: Significant Indicators from Univariate Analysis in the Thrombolysis Cohort. Table S4: Significant Indicators from Univariate Analysis in the Non‐thrombolysis Cohort. Table S5: Multivariate Regression Analysis of Differential Indicators Between the Thrombolysis and the Non‐thrombolysis Cohorts. Table S6: The Categorical Model and Corresponding Integral Metrics with their Respective Score Values. Figure S1: Variables Collected in the Study: Non‐laboratory Indicators. Figure S2: Variables Collected in the Study: Laboratory Indicators. ESR, Erythrocyte Sedimentation Rate; NT‐ProBNP, N‐Terminal Pro‐B‐Type Natriuretic Peptide; eGFR, Estimated Glomerular Filtration Rate. Figure S3: Development and Validation of the PAIST Scale for Prognostic Risk Stratification in Ischemic Stroke Patients. RCS, restricted cubic splines; PAIST Scale, Prognostic Scale of AIS Acute Stage Based on Treatment Stratification; AUC‐ROC, area under the receiver operating characteristic curve. Analyses were performed separately in thrombolysis and non‐thrombolysis cohorts. Development phase (model training sets, patients discharged between 2015 and 2020): Risk features were selected via univariate and multivariate regression (excluding collinear or insignificant variables). Continuous variables analyzed using 3‐knot RCS to identify cut‐offs (OR = 1, adjusted for covariates), then categorized and incorporated into logistic regression. Coefficients were standardized to derive the PAIST scale, which stratifies patients into low (< 10%), moderate (10%–50%), and high (> 50%) risk groups for poor prognosis. Validation phase (external validation sets, patients discharged in 2021): The predictive performance of the PAIST Scale was evaluated against a baseline NIHSS‐based [file CNS-31-e70637-s001.docx]

**Supplementary Table S1. Modified Rankin Scale (mRS) Grades and Corresponding Disability Levels.**

| Grade | mRS Description |
| --- | --- |
| 0 | No symptoms at all |
| 1 | No significant disability: despite symptoms, able to carry out all usual duties and activities |
| 2 | Slight disability: unable to perform all previous activities but able to look after own affairs without assistance |
| 3 | Moderate disability: requiring some help but able to walk without assistance |
| 4 | Moderately severe disability: unable to walk without assistance and unable to attend to own bodily needs without assistance |
| 5 | Severe disability: bedridden, incontinent and requiring constant nursing care and attention |
| 6 | Death |

Adapted from [Banks JL, Marotta CA. Outcomes validity and reliability of the modified Rankin scale: implications for stroke clinical trials: a literature review and synthesis. *Stroke*. 2007;38(3):1091-1096.], with modifications.

**Supplementary Table S2. Comparison of Baseline Characteristics Between Thrombolysis and Non-Thrombolysis Cohorts Across Training and External Validation Datasets**

|  | **Non-thrombolysis Cohort** | | | | **Thrombolysis Cohort** | | | | **P Value Between Non-Thrombolytic and Thrombolysis Cohort** |
| --- | --- | --- | --- | --- | --- | --- | --- | --- | --- |
|  | **All (N=1534)** | **Train (N=1198)** | **Validation (N=336)** | ***P* Value  Train vs. Validation** | **All (N=437)** | **Train (N=357)** | **Validation (N=80)** | ***P* value  Train vs. Validation** |  |
| ***Outcome event, n(%)*** | | | | | | | | | |
| mRS at Discharge >2 | 463 (30.2%) | 380(31.7%) | 83 (24.7%) | 0.016 | 151(34.6%) | 121(33.9%) | 30(37.5%) | 0.629 | 0.093 |
| Mortality Count | 27 (1.76%) | 24 (2.00%) | 3 (0.89%) | 0.257 | 19 (4.35%) | 17 (4.76%) | 2 (2.50%) | 0.547 | 0.003 |
| ***General data, Mean (SD)*** | | | | | | | | | |
| Number of Male (%) | 1128(73.5%) | 876(73.1%) | 252(75.0%) | 0.535 | 301(68.9%) | 246(68.9%) | 55(68.8%) | 1 | 0.063 |
| Age (year) | 63.3 (13.1) | 63.0 (13.1) | 64.3 (13.0) | 0.124 | 66.8 (11.8) | 66.9 (11.8) | 66.1 (11.8) | 0.58 | <0.001 |
| BMI（kg/m²） | 26.0 (17.1) | 25.8 (17.8) | 26.8 (14.2) | 0.268 | 25.8 (15.8) | 26.1 (17.4) | 24.2 (1.99) | 0.042 | 0.768 |
| Days from Onset to Hospital Admission | 1.58 (0.80) | 1.53 (0.81) | 1.76 (0.76) | <0.001 | 0.32 (0.48) | 0.30 (0.48) | 0.39 (0.47) | 0.117 | <0.001 |
| SBP on Admission（mmHg) | 151 (22.7) | 150 (22.5) | 151 (23.5) | 0.775 | 152 (22.4) | 152 (22.8) | 152 (20.7) | 0.874 | 0.322 |
| DBP on Admission（mmHg) | 84.9 (12.3) | 85.0 (12.1) | 84.7 (13.0) | 0.67 | 82.4 (12.9) | 82.0 (12.5) | 83.9 (14.5) | 0.296 | <0.001 |
| Individual with DVT (%) | 463 (30.2%) | 373(31.1%) | 90 (26.8%) | 0.142 | 136 (31.1%) | 114 (31.9%) | 22 (27.5%) | 0.522 | 0.751 |
| ***Medical History, n (%)*** | | | | | | | | | |
| Antidiabetic Medication Use | 301 (19.6%) | 213 (17.8%) | 88 (26.2%) | 0.001 | 102 (23.3%) | 77 (21.6%) | 25 (31.2%) | 0.088 | 0.102 |
| Antihypertensive Medication Use | 722 (47.1%) | 559 (46.7%) | 163 (48.5%) | 0.59 | 251 (57.4%) | 208 (58.3%) | 43 (53.8%) | 0.54 | <0.001 |
| Lipid-Lowering Medication Use | 189 (12.3%) | 124 (10.4%) | 65 (19.3%) | <0.001 | 59 (13.5%) | 40 (11.2%) | 19 (23.8%) | 0.005 | 0.566 |
| Previous Stroke | 103 (6.71%) | 65 (5.43%) | 38 (11.3%) | <0.001 | 34 (7.78%) | 26 (7.28%) | 8 (10.0%) | 0.556 | 0.505 |
| Previous Myocardial Infarction | 42 (2.74%) | 33 (2.75%) | 9 (2.68%) | 1 | 12 (2.75%) | 6 (1.68%) | 6 (7.50%) | 0.011 | 1 |
| History of Atrial Fibrillation | 200 (13.0%) | 164 (13.7%) | 36 (10.7%) | 0.18 | 88 (20.1%) | 71 (19.9%) | 17 (21.2%) | 0.904 | <0.001 |
| History of Hypertension | 680 (44.3%) | 511 (42.7%) | 169 (50.3%) | 0.015 | 216 (49.4%) | 169 (47.3%) | 47 (58.8%) | 0.085 | 0.067 |
| History of Diabetes Mellitus | 264 (17.2%) | 199 (16.6%) | 65 (19.3%) | 0.275 | 84 (19.2%) | 61 (17.1%) | 23 (28.7%) | 0.025 | 0.367 |
| History of Smoking | 1137(74.1%) | 930 (77.6%) | 207 (61.6%) | <0.001 | 325 (74.4%) | 281 (78.7%) | 44 (55.0%) | <0.001 | 0.965 |
| History of Alcohol Consumption | 910 (59.3%) | 783 (65.4%) | 127 (37.8%) | <0.001 | 269 (61.6%) | 245 (68.6%) | 24 (30.0%) | <0.001 | 0.432 |
| ***Laboratory Parameters Associated with the PAIST Scale,* Mean (SD)** | | | | | | | | | |
| PT (Seconds） | 11.4 (1.21) | 11.4 (1.25) | 11.6 (1.06) | 0.007 | 11.5 (0.99) | 11.6 (1.03) | 11.4 (0.82) | 0.255 | 0.014 |
| NSE (ng/ml） | 14.3 (8.05) | 14.5 (8.54) | 13.7 (5.93) | 0.06 | 14.5 (5.76) | 14.4 (5.62) | 15.1 (6.39) | 0.361 | 0.503 |
| Serum Potassium (mmol/L) | 3.76 (0.35) | 3.78 (0.36) | 3.69 (0.32) | <0.001 | 3.82 (0.39) | 3.83 (0.40) | 3.78 (0.36) | 0.247 | 0.008 |
| FBG (mmol/L) | 6.59 (2.19) | 6.59 (2.25) | 6.62 (1.96) | 0.819 | 6.58 (2.10) | 6.58 (2.16) | 6.58 (1.78) | 0.996 | 0.922 |
| NEUT% | 66.9 (10.7) | 66.6 (10.7) | 67.9 (10.9) | 0.042 | 69.7 (11.3) | 70.2 (11.3) | 67.7 (10.9) | 0.066 | <0.001 |
| ***Baseline NIHSS Score, n(%)*** | | | | | | | | | <0.001 |
| Mild Stroke (NHISS ≤ 4) | 893 (58.2%) | 680 (56.8%) | 213 (63.4%) | 0.082 | 167 (38.2%) | 131 (36.7%) | 36 (45.0%) | 0.385 |  |
| Moderate Stroke (NHISS 5 - 15) | 548 (35.7%) | 441 (36.8%) | 107 (31.8%) |  | 227 (51.9%) | 190 (53.2%) | 37 (46.2%) |  |  |
| Severe Stroke (NHISS ≥ 16) | 93 (6.06%) | 77 (6.43%) | 16 (4.76%) |  | 43 (9.84%) | 36 (10.1%) | 7 (8.75%) |  |  |
| ***OCSP, n (%)*** | | | | | | | | | <0.001 |
| LACI | 210 (13.7%) | 159 (13.3%) | 51 (15.2%) | 0.673 | 57 (13.0%) | 45 (12.6%) | 12 (15.0%) | 0.882 |  |
| PACI | 784 (51.1%) | 616 (51.4%) | 168 (50.0%) |  | 260 (59.5%) | 214 (59.9%) | 46 (57.5%) |  |  |
| POCI | 461 (30.1%) | 364 (30.4%) | 97 (28.9%) |  | 87 (19.9%) | 70 (19.6%) | 17 (21.2%) |  |  |
| TACI | 79 (5.15%) | 59 (4.92%) | 20 (5.95%) |  | 33 (7.55%) | 28 (7.84%) | 5 (6.25%) |  |  |
| ***TOAST, n(%)*** | | | | | | | | | <0.001 |
| SAO | 446 (29.1%) | 325 (27.1%) | 121 (36.0%) | 0.03 | 100 (22.9%) | 81 (22.7%) | 19 (23.8%) | 0.185 |  |
| LAA | 578 (37.7%) | 464 (38.7%) | 114 (33.9%) |  | 158 (36.2%) | 136 (38.1%) | 22 (27.5%) |  |  |
| CE | 193 (12.6%) | 158 (13.2%) | 35 (10.4%) |  | 86 (19.7%) | 69 (19.3%) | 17 (21.2%) |  |  |
| SOE | 48 (3.13%) | 37 (3.09%) | 11 (3.27%) |  | 7 (1.60%) | 4 (1.12%) | 3 (3.75%) |  |  |
| SUE | 269 (17.5%) | 214 (17.9%) | 55 (16.4%) |  | 86 (19.7%) | 67 (18.8%) | 19 (23.8%) |  |  |

mRS, Modified Rankin Scale; BMI, Body Mass Index; SBP, Systolic Blood Pressure; DBP, Diastolic Blood Pressure; DVT, Deep Venous Thrombosis; PAIST Scale, Prognostic Scale of AIS Acute Stage Based on Treatment Stratification; PT Prothrombin time; NSE, Neuron Specific Enolase; NEUT%, Neutrophil Percentage; FBG, Fasting Blood Glucose ; NIHSS, National Institutes of Health Stroke Scale; OCSP, Oxfordshire Community Stroke Project; LACI, Lacunar Infarct; PAIC, Partial Anterior Circulation Infarct; POCI, Posterior Circulation Infarct; TACI, Total Anterior Circulation Infarct; TOAST, Trial of Org 10172 in Acute Stroke Treatment; SAO, Small Artery Occlusion; LAA, Large-artery Atherosclerosis; CE, Cardioembolism; SOE, Other Determined Etiology; SUE, Undetermined Etiology.

**Supplementary Table S3.** **Significant Indicators from Univariate Analysis in the Thrombolysis Cohort**

|  | **Estimate** | **P** | **OR** | **CI lower** | **CI upper** |
| --- | --- | --- | --- | --- | --- |
| Gender (Male） | -0.647 | 0.006 | 0.524 | 0.329 | 0.834 |
| Absolute Lymphocyte Count | -0.473 | 0.009 | 0.623 | 0.432 | 0.879 |
| Lymphocyte Percentage | -0.058 | <0.001 | 0.944 | 0.918 | 0.968 |
| PLR | 0.005 | 0.001 | 1.005 | 1.002 | 1.008 |
| CRP | 0.016 | 0.005 | 1.016 | 1.006 | 1.029 |
| Age | 0.026 | 0.009 | 1.026 | 1.007 | 1.046 |
| ESR | 0.038 | <0.001 | 1.039 | 1.019 | 1.061 |
| NEUT% | 0.049 | 0.000 | 1.050 | 1.028 | 1.073 |
| NLRs | 0.071 | 0.004 | 1.074 | 1.026 | 1.132 |
| NSE | 0.107 | <0.001 | 1.112 | 1.062 | 1.174 |
| WBC | 0.128 | 0.002 | 1.137 | 1.048 | 1.237 |
| Absolute Neutrophil Count | 0.159 | <0.001 | 1.172 | 1.079 | 1.280 |
| FBG | 0.170 | 0.001 | 1.185 | 1.072 | 1.315 |
| HDL-C | 0.842 | 0.033 | 2.321 | 1.071 | 5.082 |
| Presence of Atrial Fibrillation | 0.889 | 0.001 | 2.432 | 1.432 | 4.144 |
| TOAST_SUE | 1.441 | 0.001 | 4.226 | 1.897 | 10.042 |
| Presence of DVT | 1.463 | <0.001 | 4.318 | 2.698 | 6.979 |
| TOAST_SAO | 1.481 | <0.001 | 4.395 | 2.159 | 9.748 |
| TOAST_CE | 1.815 | <0.001 | 6.141 | 2.804 | 14.453 |
| OCSP_POCI | 1.924 | 0.003 | 6.851 | 2.185 | 30.353 |
| OCSP_PACI | 2.043 | 0.001 | 7.710 | 2.690 | 32.564 |
| Mild to Moderate Stroke  (Baseline NIHSS 5-15) | 2.331 | <0.001 | 10.292 | 5.178 | 22.901 |
| OCSP_LACI | 3.386 | <0.001 | 29.556 | 8.096 | 146.909 |
| Severe Stroke  (Baseline NIHSS≥16) | 4.216 | <0.001 | 67.778 | 23.991 | 224.302 |

PLR, Platelet-to-Lymphocyte Ratio; CRP, C-Reactive Protein; ESR, Erythrocyte Sedimentation Rate; NEUT%, Neutrophil Percentage; NLRs, Neutrophil-to-Lymphocyte Ratio; NSE, Neuron-Specific Enolase; WBC, White Blood Cell Count; FBG, Fasting Blood Glucose; HDL-C, High-Density Lipoprotein Cholesterol; DVT, Deep Vein Thrombosis; NIHSS, National Institutes of Health Stroke Scale; OCSP, Oxfordshire Community Stroke Project; LACI, Lacunar Infarct; PAIC, Partial Anterior Circulation Infarct; POCI, Posterior Circulation Infarct; TOAST, Trial of Org 10172 in Acute Stroke Treatment; SAO, Small Artery Occlusion; CE, Cardioembolism; SUE, Undetermined Etiology.

**Supplementary Table S4. Significant Indicators from Univariate Analysis in the Non-Thrombolysis Cohort**

|  | **Estimate** | **P** | **OR** | **CI Lower** | **CI Upper** |
| --- | --- | --- | --- | --- | --- |
| Serum Uric Acid | -1.504 | 0.021 | 0.222 | 0.061 | 0.785 |
| Absolute Lymphocyte Count | -0.659 | 0.000 | 0.517 | 0.418 | 0.635 |
| Serum Potassium | -0.489 | 0.005 | 0.613 | 0.434 | 0.862 |
| Monocyte Percentage | -0.088 | 0.005 | 0.916 | 0.861 | 0.972 |
| Lymphocyte Percentage | -0.081 | <0.001 | 0.923 | 0.908 | 0.937 |
| Platelet Count | -0.002 | 0.026 | 0.998 | 0.996 | 1.000 |
| NT-proBNP | 0.000 | 0.001 | 1.000 | 1.000 | 1.000 |
| PLR | 0.006 | <0.001 | 1.006 | 1.004 | 1.008 |
| Serum Homocysteine | 0.020 | 0.009 | 1.020 | 1.005 | 1.036 |
| Age | 0.026 | <0.001 | 1.026 | 1.016 | 1.037 |
| ESR | 0.032 | <0.001 | 1.032 | 1.023 | 1.042 |
| CRP | 0.040 | <0.001 | 1.040 | 1.031 | 1.051 |
| NSE | 0.059 | <0.001 | 1.060 | 1.041 | 1.082 |
| NEUT% | 0.070 | <0.001 | 1.072 | 1.058 | 1.086 |
| FBG | 0.145 | <0.001 | 1.156 | 1.097 | 1.219 |
| BUN | 0.146 | <0.001 | 1.157 | 1.092 | 1.231 |
| PT | 0.163 | 0.002 | 1.177 | 1.066 | 1.314 |
| D-Dimer | 0.166 | <0.001 | 1.181 | 1.097 | 1.294 |
| WBC | 0.183 | <0.001 | 1.201 | 1.145 | 1.262 |
| NLRs | 0.192 | <0.001 | 1.212 | 1.157 | 1.273 |
| Absolute Neutrophil Count | 0.239 | <0.001 | 1.270 | 1.205 | 1.340 |
| History of Hypertension | 0.281 | 0.025 | 1.324 | 1.036 | 1.692 |
| Antihypertensive Medication Use | 0.367 | 0.003 | 1.443 | 1.130 | 1.843 |
| Fibrinogen Quantification | 0.436 | <0.001 | 1.547 | 1.351 | 1.777 |
| Essen_ Moderate Risk | 0.533 | <0.001 | 1.704 | 1.322 | 2.194 |
| TOAST_SUE | 0.561 | 0.008 | 1.752 | 1.159 | 2.651 |
| OCSP_POCI | 0.563 | 0.022 | 1.755 | 1.098 | 2.887 |
| Presence of Atrial Fibrillation | 0.583 | 0.001 | 1.791 | 1.277 | 2.505 |
| History of Previous Stroke | 0.914 | <0.001 | 2.495 | 1.508 | 4.141 |
| OCSP_PACI | 0.959 | <0.001 | 2.608 | 1.685 | 4.176 |
| TOAST_SOE | 1.030 | 0.005 | 2.802 | 1.335 | 5.720 |
| TOAST_SAO | 1.089 | <0.001 | 2.971 | 2.127 | 4.198 |
| TOAST_CE | 1.221 | <0.001 | 3.389 | 2.221 | 5.197 |
| Presence of DVT | 1.750 | <0.001 | 5.756 | 4.410 | 7.542 |
| INR | 1.876 | 0.002 | 6.530 | 2.089 | 23.568 |
| Mild to Moderate Stroke  (Baseline NIHSS 5-15) | 2.995 | <0.001 | 19.985 | 14.147 | 28.777 |
| TNT | 3.372 | 0.032 | 29.146 | 2.259 | 1011.461 |
| OCSP_LACI | 3.811 | <0.001 | 45.186 | 18.845 | 127.568 |
| Severe Stroke  (Baseline NIHSS≥16) | 5.528 | <0.001 | 251.533 | 98.971 | 852.612 |
| Urine Specific Gravity | 35.015 | <0.001 | 1.61E+15 | 1.04E+09 | 2.97E+21 |

NT-proBNP, N-terminal pro-B-type Natriuretic Peptide; PLR, Platelet-to-Lymphocyte Ratio; ESR, Erythrocyte Sedimentation Rate; CRP, C-Reactive Protein; NSE, Neuron-Specific Enolase; NEUT%, Neutrophil Percentage; FBG, Fasting Blood Glucose; BUN, Blood Urea Nitrogen; PT, Prothrombin Time; WBC, White Blood Cell Count; NLRs, Neutrophil-to-Lymphocyte Ratio; DVT, Deep Vein Thrombosis; INR, International Normalized Ratio; Essen_ Moderate Risk, Essen Stroke Risk Score 3-6; TNT, Troponin T; NIHSS, National Institutes of Health Stroke Scale; OCSP, Oxfordshire Community Stroke Project; LACI, Lacunar Infarct; PAIC, Partial Anterior Circulation Infarct; POCI, Posterior Circulation Infarct; TOAST, Trial of Org 10172 in Acute Stroke Treatment; SAO, Small Artery Occlusion; CE, Cardioembolism; SOE, Other Determined Etiology; SUE, Undetermined Etiology.

**Supplementary Table S5. Multivariate Regression Analysis of Differential Indicators Between the Thrombolysis and the Non-thrombolysis Cohorts.**

|  | Estimate | Std.Error | z value | Pr(>\|z\|) |
| --- | --- | --- | --- | --- |
| ***Thrombolysis Cohort*** |  |  |  |  |
| NEUT% | 0.037 | 0.019 | 1.914 | 0.056 |
| NSE | 0.059 | 0.027 | 2.176 | 0.030 |
| Presence of DVT | 0.794 | 0.289 | 2.750 | 0.006 |
| Mild to Moderate Stroke (Baseline NIHSS 5-15) | 2.039 | 0.400 | 5.100 | <0.001 |
| Severe Stroke (Baseline NIHSS≥16) | 3.481 | 0.590 | 5.897 | <0.001 |
| ***Non-thrombolysis Cohort*** |  |  |  |  |
| Serum Potassium | -0.453 | 0.239 | -1.894 | 0.058 |
| PT | -0.157 | 0.084 | -1.863 | 0.062 |
| Age | 0.019 | 0.007 | 2.783 | 0.005 |
| NSE | 0.020 | 0.010 | 2.024 | 0.043 |
| NEUT% | 0.056 | 0.013 | 4.342 | 0.000 |
| FBG | 0.087 | 0.038 | 2.325 | 0.020 |
| Presence of DVT | 0.999 | 0.182 | 5.478 | 0.000 |
| Mild to Moderate Stroke (Baseline NIHSS 5-15) | 2.823 | 0.191 | 14.749 | <0.001 |
| Severe Stroke (Baseline NIHSS≥16) | 4.598 | 0.556 | 8.270 | < 0.001 |

NEUT%, Neutrophil Percentage; NSE, Neuron-specific enolase; DVT, Deep Vein Thrombosis; PT, Prothrombin Time; FBG, Fasting Blood Glucose.

**Supplementary Table S6. The Categorical Model and Corresponding Integral Metrics with Their Respective Score Values**

| Parameters of the  Non-Thrombolysis Patients | Score  Value | Rounding Coefficients |  | Parameters of the  Thrombolysis Patients | Score  Value | Rounding Coefficients |
| --- | --- | --- | --- | --- | --- | --- |
| Baseline NIHSS Score | | |  | Baseline NIHSS Score | | |
| <5 | 0 | 0 |  | <5 | 0 | 0 |
| 5-15 | 61 | 12.2 (12) |  | 5-15 | 56 | 11.2 (11) |
| ≥16 | 100 | 20 (20) |  | ≥16 | 100 | 20 (20) |
| Presence of DVT |  |  |  | Presence of DVT | |  |
| No | 0 | 0 |  | No | 0 | 0 |
| Yes | 22 | 4.4 (4) |  | Yes | 23 | 4.6 (5) |
| NSE > 12.5 ng/ml |  |  |  | NSE > 13 ng/ml | |  |
| No | 0 | 0 |  | No | 0 | 0 |
| Yes | 4 | 0.8 (1) |  | Yes | 14 | 2.8 (3) |
| NEUT% > 66% |  |  |  | NEUT% > 70% | |  |
| No | 0 | 0 |  | No | 0 | 0 |
| Yes | 16 | 3.2 (3) |  | Yes | 23 | 4.6 (5) |
| Age > 64 years |  |  |  |  |  |  |
| No | 0 | 0 |  |  |  |  |
| Yes | 8 | 1.6 (2) |  |  |  |  |
| PT < 11.2 seconds |  |  |  |  |  |  |
| No | 0 | 0 |  |  |  |  |
| Yes | 1 | 0.2 (0) |  |  |  |  |
| FBG > 5.8 mmol/l |  |  |  |  |  |  |
| No | 0 | 0 |  |  |  |  |
| Yes | 11 | 2.2 (2) |  |  |  |  |
| K^+^ < 3.8 mmol/l |  |  |  |  |  |  |
| No | 0 | 0 |  |  |  |  |
| Yes | 5 | 1 (1) |  |  |  |  |

Rounding Coefficients: each variable's coefficient in the categorical model was divided by 5, rounded to the nearest whole number (values in parentheses represent the final rounded scores).

DVT, Deep Vein Thrombosis; NSE, Neuron Specific Enolase; NEUT%, Neutrophil Percentage; PT, Prothrombin Time; FBG, Fasting Blood Glucose; K^+^, Serum Potassium.


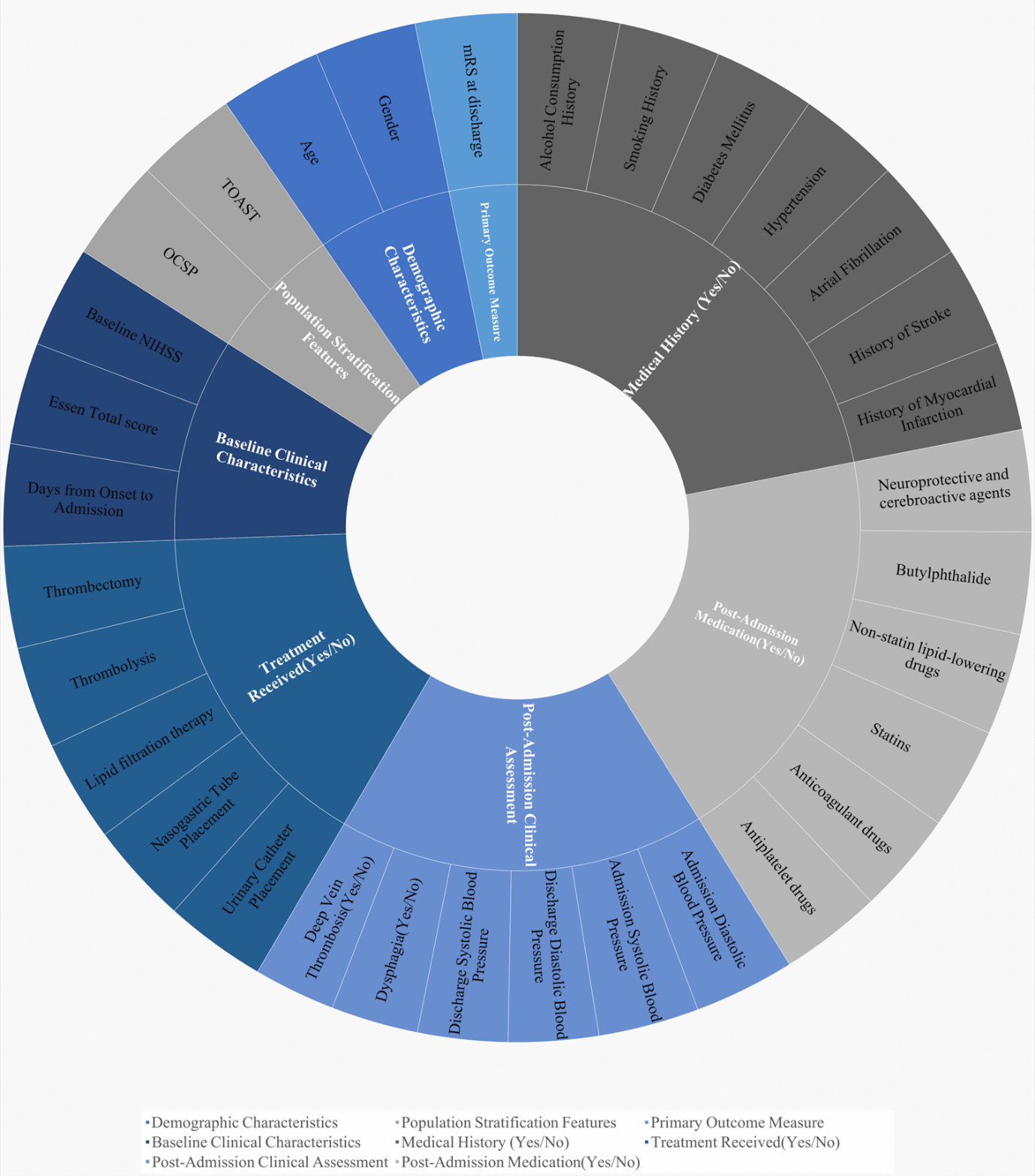


**Supplementary Figure S1. Variables Collected in the Study: Non-laboratory Indicators.**


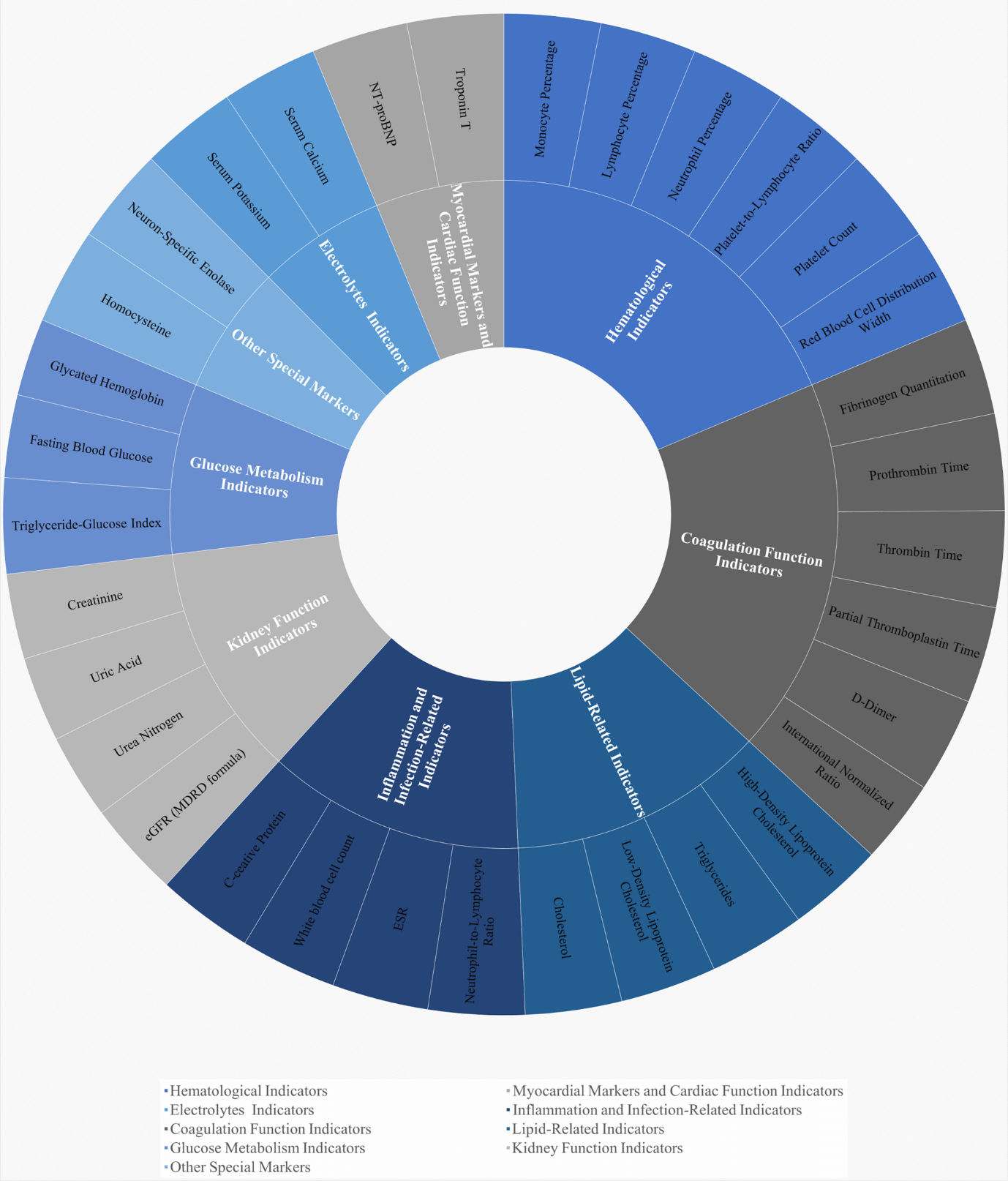


**Supplementary Figure S2. Variables Collected in the Study: Laboratory Indicators.** ESR, Erythrocyte Sedimentation Rate; NT-ProBNP, N-Terminal Pro-B-Type Natriuretic Peptide; eGFR, Estimated Glomerular Filtration Rate.

**
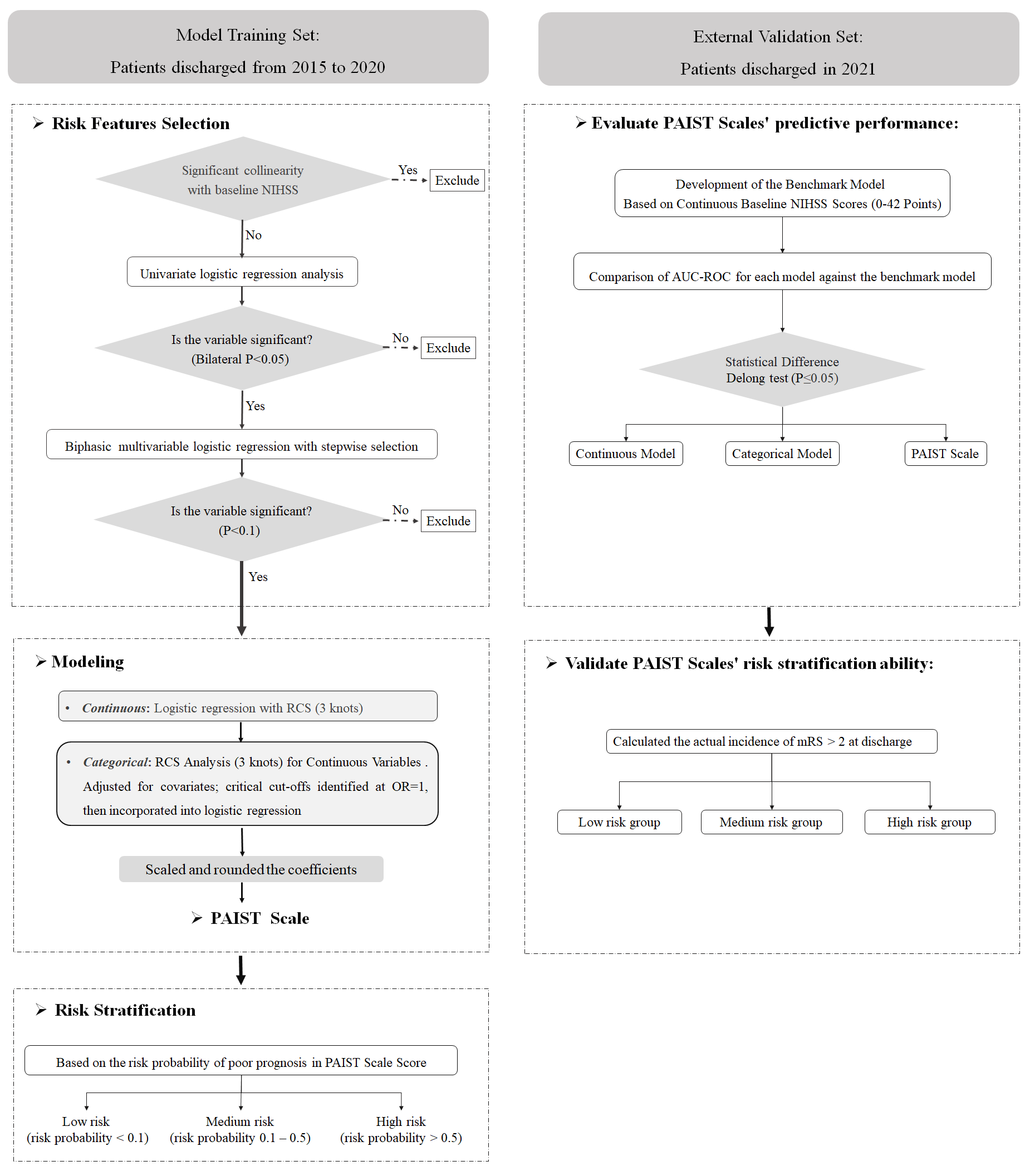
**

**Supplementary Figure S3. Development and Validation of the PAIST Scale for Prognostic Risk Stratification in Ischemic Stroke Patients.** RCS, restricted cubic splines; PAIST Scale, Prognostic Scale of AIS Acute Stage Based on Treatment Stratification; AUC-ROC, area under the receiver operating characteristic curve. Analyses were performed separately in thrombolysis and non-thrombolysis cohorts. Development phase (model training sets, patients discharged between 2015–2020): Risk features were selected via univariate and multivariate regression (excluding collinear or insignificant variables). Continuous variables analyzed using 3-knot RCS to identify cut-offs (OR = 1, adjusted for covariates), then categorized and incorporated into logistic regression. Coefficients were standardized to derive the PAIST scale, which stratifies patients into low (< 10%), medium (10–50%), and high (> 50%) risk groups for poor prognosis. Validation phase (external validation sets, patients discharged in 2021): The predictive performance of the PAIST Scale was evaluated against a baseline NIHSS-based benchmark model (via DeLong test for AUC-ROC comparison), and its risk stratification was further validated.

**
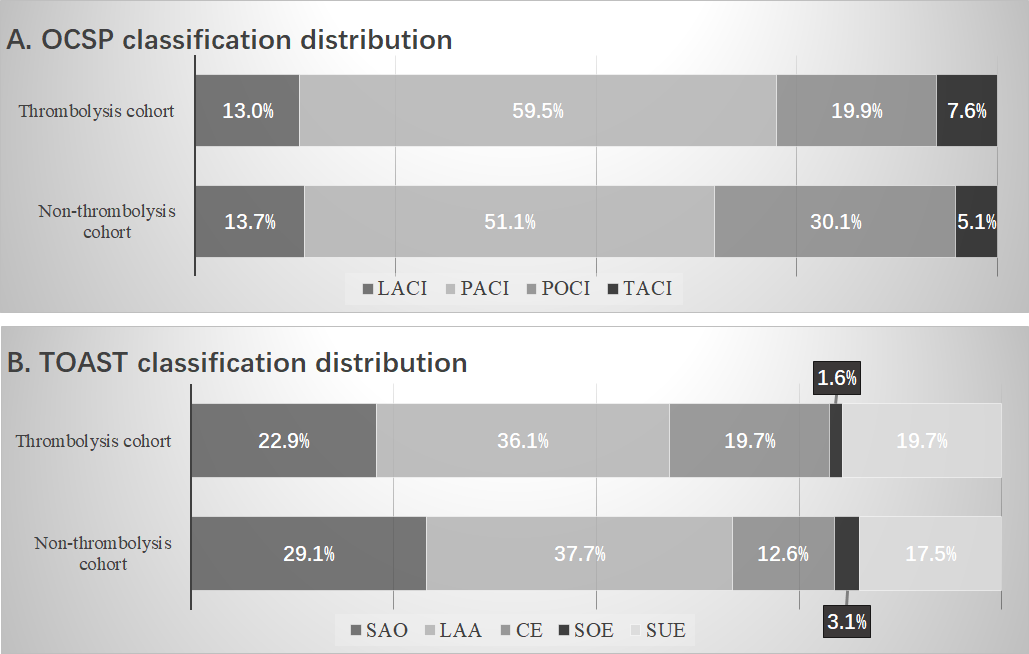
**

**Supplementary Figure S4. Distribution of Stroke Subtypes in Subgroup Populations.**

(A) OCSP, Oxfordshire Community Stroke Project; LACI, Lacunar Infarct; PAIC, Partial Anterior Circulation Infarct; POCI, Posterior Circulation Infarct; TACI, Total Anterior Circulation Infarct; (B) TOAST, Trial of Org 10172 in Acute Stroke Treatment; SAO, Small Artery Occlusion; LAA, Large-Artery Atherosclerosis; CE, Cardioembolism; SOE, Other Determined Etiology; SUE, Undetermined Etiology.


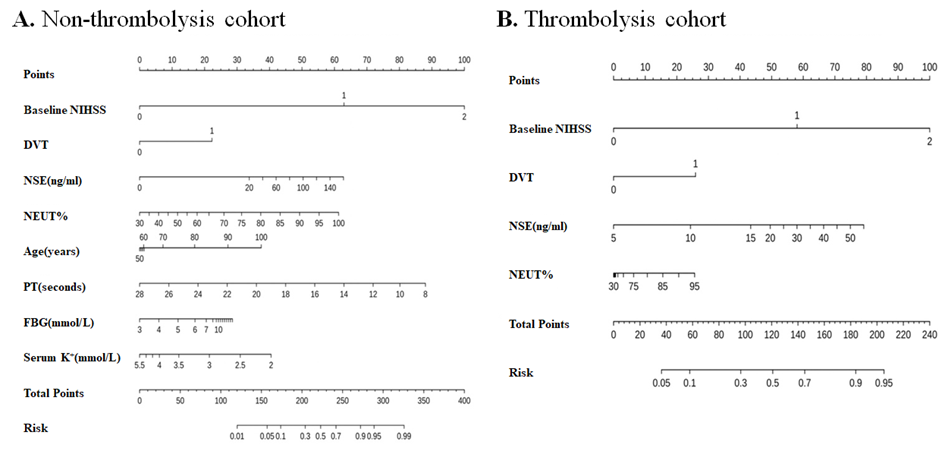


**Supplementary Figure S5. Nomograms of Continuous Models for Predicting Poor Prognosis Risk in AIS.** Variables included in the nomograms and their definitions: Baseline NIHSS (0 = NIHSS ≤ 4; 1 = NIHSS 5-15; 2 = NIHSS ≥ 16); DVT (deep vein thrombosis, 0 = No, 1 = Yes); NIHSS, National Institutes of Health Stroke Scale; NSE, Neuron Specific Enolase; NEUT%, Neutrophil Percentage; PT, Prothrombin Time; FBG, Fasting Blood Glucose; Serum K^+^, Serum Potassium. Each variable is assigned points value, and the total points correspond to both a predicted risk probability (on a 0–10 scale) and an absolute risk (0–100%). The bottom axis indicates risk stratification from low to high.


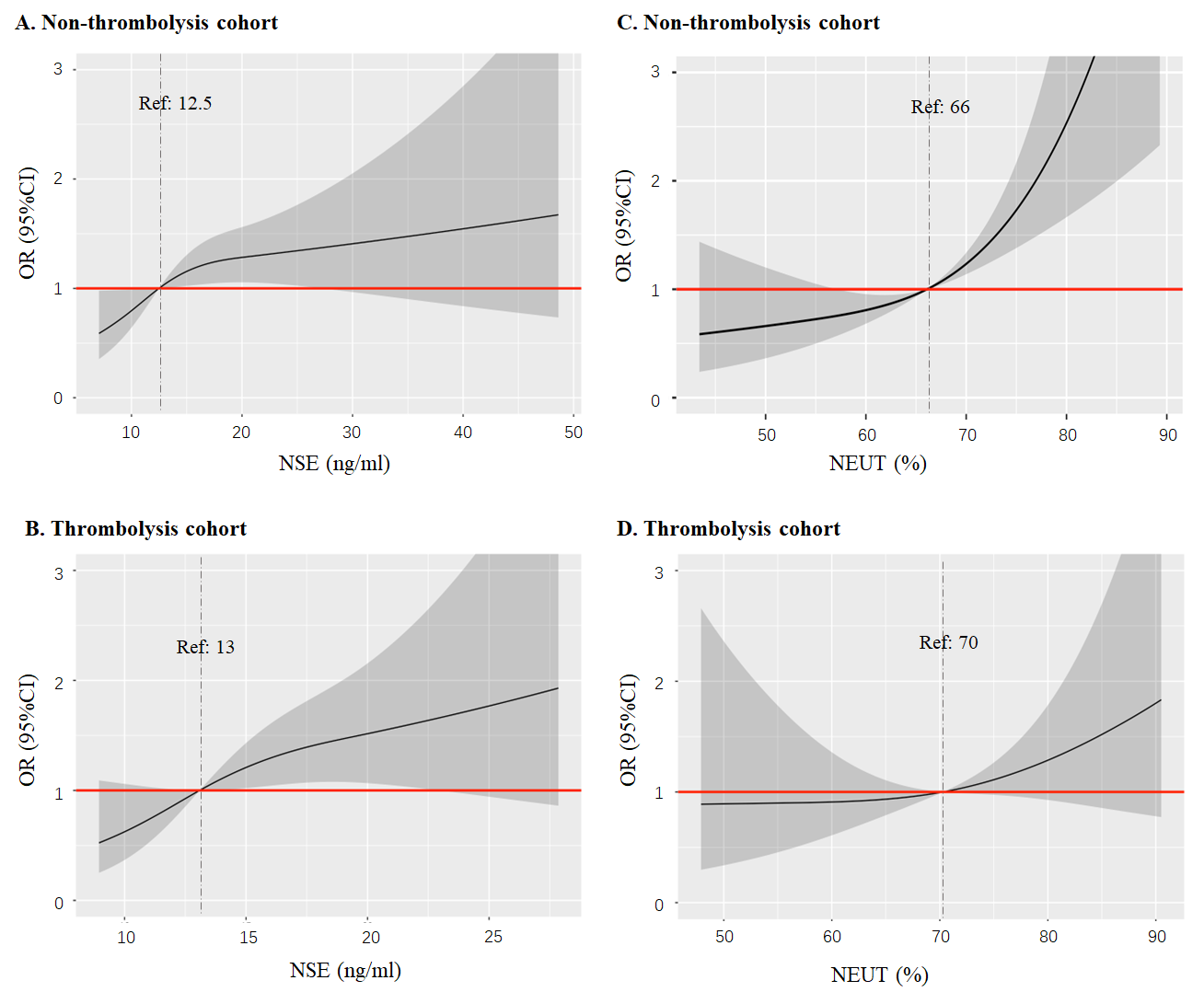


**Supplementary Figure S6. Consistent Nonlinear Associations of Clinical Indicators with Poor Stroke Outcomes Across Two Cohorts.** This figure presents associations analyzed using restricted cubic spline with 3 knots. OR, odds ratio; CI, confidence interval; NIHSS, National Institutes of Health Stroke Scale; DVT, deep vein thrombosis; NSE, neuron-specific enolase; NEUT%, Neutrophil Percentage; PT, Prothrombin Time; FBG, Fasting Blood Glucose; K⁺, Serum Potassium, Ref, Reference Value. Due to inherent differences in data distribution, x-axis scales vary between cohorts; thus, interpretation should focus on curve-derived association trends rather than absolute numerical positions. Reference thresholds were determined via restricted cubic splines analysis, identifying inflection points where OR = 1. OR estimates were adjusted for covariates: non-thrombolysis cohort (baseline NIHSS ≤ 4, DVT-free, NSE = 12.5 ng/ml, NEUT%=66%, PT = 11 s, FBG = 5.8 mmol/L, K⁺ = 3.8 mmol/L, age = 64 years) and thrombolysis cohort (baseline NIHSS 5-15, DVT-free, NSE = 13 ng/ml, NEUT% = 70%), with analyzed variables excluded. Solid red line: Reference line (OR = 1); Gray shaded area: 95% confidence interval of the estimated OR; Dashed vertical line: cutoff value where OR = 1.
